# Effect of Age of *Agave tequilana* Weber Blue Variety on Quality and Authenticity Parameters for the Tequila 100% Agave Silver Class: Evaluation at the Industrial Scale Level

**DOI:** 10.3390/foods10123103

**Published:** 2021-12-14

**Authors:** Efraín Acosta-Salazar, Rocío Fonseca-Aguiñaga, Walter M. Warren-Vega, Ana I. Zárate-Guzmán, Marco A. Zárate-Navarro, Luis A. Romero-Cano, Armando Campos-Rodríguez

**Affiliations:** 1 Departamento de Ciencias Biotecnológicas y Ambientales, Universidad Autónoma de Guadalajara, Av. Patria 1201, Zapopan 45129, Mexico; efrain.acosta@edu.uag.mx (E.A.-S.); marco.zarate@edu.uag.mx (M.A.Z.-N.); 2 Grupo de Investigación en Materiales y Fenómenos de Superficie, Departamento de Ciencias Biotecnológicas y Ambientales, Universidad Autónoma de Guadalajara, Av. Patria 1201, Zapopan 45129, Mexico; rfonseca@crt.org.mx (R.F.-A.); wm.warren@edu.uag.mx (W.M.W.-V.); ana.zarate@edu.uag.mx (A.I.Z.-G.); 3 Laboratorio de Isotopía, Consejo Regulador del Tequila A. C., Av. Patria 723, Zapopan 45030, Mexico; 4 Centro de Investigación y Estudios de Posgrado, Facultad de Ciencias Químicas, Universidad Autónoma de San Luis Potosí, San Luis Potosí 78060, Mexico

**Keywords:** agave age, tequila, quality and authenticity, higher alcohols, industrial scale tequila production, agave exudate

## Abstract

Due to the oversupply and scarcity cycles of the *Agave tequilana* Weber blue variety, the effect of agave age (harvested in 4, 5, and 6 years) as raw material for the tequila 100% agave silver class was studied for each stage in a full-scale (industrial) process. Harvested plants showed differences in their morphological characteristics that affected the amount of juice; this had an impact in the fermentation stage since must composition was modified in the nitrogen content and juice/exudate ratio. This was noticed due to an increase in the production of higher alcohols attributed to the odd-chain fatty of the exudate, which affects n-propanol production. The characterization of the final product showed the feasibility to use agaves (less than 7 years) to produce the Tequila 100% agave silver class and to comply with the quality criteria. Furthermore, the final product was analyzed with the gas chromatography-isotope ratio mass-spectrometry technique to determine its authenticity. The δ^13^C_VPDB_ isotopic parameter (−13.40‰ in average) values show the type of plant used as a raw material for ethanol production, while the δ^18^O_VSMOW_ (20.52‰ in average) isotopic parameter can be helpful in corroborating and ensuring the traceability of the product and the geographical location of the beverage production.

## 1. Introduction

Tequila is a representative distilled beverage of Mexico currently attracting international consumers for its unique organoleptic properties. Their production is thoroughly controlled, starting with the registration of the *Agave tequilana* Weber blue variety plantations. The process continues with the agave harvest (“jima”) and its transformation into tequila through cooking, fermentation, and distillation, among other operations, where the final product is finally marketed. According to the Mexican Official Standard NOM-006-SCFI-2012, tequila is an alcoholic beverage obtained from the hearts of the *Agave tequilana* Weber blue variety, which is cultivated within the specific geographical Denomination of Origin Tequila (DOT), located within the state of Jalisco (125 municipalities), Michoacan (30 municipalities), Nayarit (8 municipalities), Guanajuato (7 municipalities), and Tamaulipas (11 municipalities). Two categories of tequila are distinguished: (a) tequila 100% agave, which uses 100% sugars from the *Agave tequilana* Weber blue variety and (b) tequila, which corresponds to an alcoholic beverage in which 51% of the sugars are from the *Agave tequilana* Weber blue variety while the other 49% of the sugars may come from other sources. Likewise, according to the characteristics of the maturation process, five classes can be defined: (a) “*Blanco*” (Silver), a transparent but not necessarily colorless product without additives obtained through distillation in which the commercial alcohol content must be adjusted by dilution with water; (b) “*Joven u Oro*” (Gold), a product that results from blending silver tequila with additives allowed by the official standard or from the mix of silver tequila with aged, extra-aged, and/or ultra-aged tequilas; (c) “*Reposado*” (aged), a product that may be enhanced by mellowing, subject to an aging process of at least two months in direct contact with the wood from oak or encino casks; (d) “*Añejo*” (extra-aged), a product that may be enhanced by mellowing in an aging process for at least one year in the wood of oak or encino recipients with V ≤ 600 L; and (e) “*Extra añejo*” (ultra-aged), a product that may be enhanced by mellowing in an aging process for at least three years in the wood of oak or encino recipients with V ≤ 600 L.

**Figure 1:**
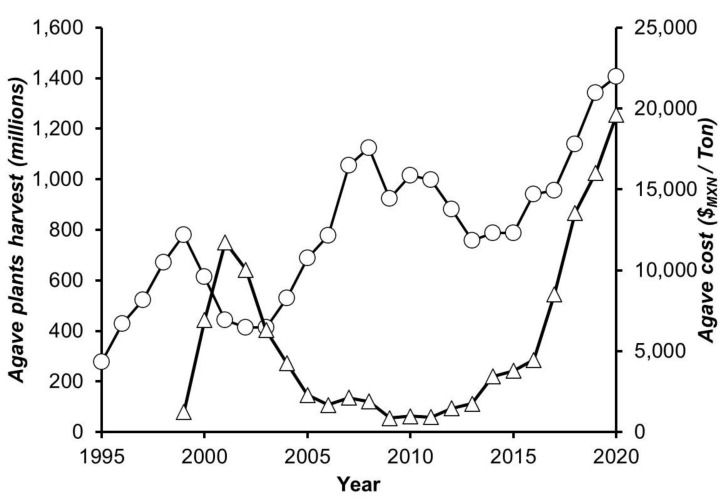
Harvest and cost of the *Agave tequilana* Weber blue variety for tequila production (jima): ○ agave plants harvest; ∆ agave cost. Data provided by CRT.

In terms of export, the tequila industry had an 18.5% increase in 2019 with respect to the previous year, with over 1874 million USD in revenue [1]. Nonetheless, the increasing demand for this beverage and the commercial expansion in the international markets have caused a particular scarcity of *Agave tequilana* Weber blue variety, the raw material for tequila production. With a periodic phenomenon that occurs every 7 or 8 years due to the time it takes an agave to maturate for its subsequent harvest [2], usually, the agave is harvested at this point since it corresponds to the time just before flowering, an optimal age when the sugar content is at its maximum in the plant [3]. According to the data from the Tequila Regulatory Council (CRT for its acronym in Spanish), in 1995 to 2012, there was a transition between agave oversupply to scarcity and then oversupply, which impacted the price of the raw material. This was related to the development of the agave plant by different stages: during the first stage (from 1 to 3 years), the plant generates its fundamental structure for its subsequent growth; during the next three years, it has a considerable increase in size and the storage of sugars starts; and from the seventh year, it begins with its reproductive phase through flowering, which reduces the concentration of sugars (unwanted event) and thus ends its life cycle. Due to the above, the *Agave tequilana* Weber blue variety presents a cyclical phenomenon of abundance and scarcity in terms of mature plants. This phenomenon is mainly due to the long time that the agave requires from its plantation to its maturity, at the variable cost per ton, and to the gradual increase in demand for agave (see Figure 1), caused by the increase in global tequila production according to data reported by the CRT. Therefore, several cases of counterfeit and adulterated beverages have been detected and documented [4], highlighting the existence of unfair producers that use agave plants from outside the Designation of Origin of Tequila (DOT) or the use of raw materials that do not meet the requirements that established the Mexican Official Standard [5]. To avoid falling into these unfair practices and to guarantee the quality standards of the beverage, several companies have started to use younger agave plants (aged between 4 to 6 years) in their processes. However, this practice could alter the overall product characteristics. Previous studies [6,7,8,9,10,11] have shown that the composition of the agave plants is affected by its age at harvest; therefore, it is suspected that its use could affect quality parameters in tequila, as previously reported with other beverages where the physicochemical and organoleptic properties are straightforwardly related to the raw material used for the must [12,13,14]. Currently, the research on the agave age effect on tequila production only has been focused in a single stage of the process, namely in the fermentation stage [15]; however, it is known that each production stage has a different influence on the generation of volatile compounds in tequila, such as higher alcohols, esters, Maillard compounds, and furfural, which impact the final product quality [16,17,18,19].

These reasons are the motivation of this contribution, where the effect of agave age was evaluated in the entire production process of the tequila 100% agave silver class at an industrial scale, evaluating the consequences of this variable in the production stages—harvest, hydrolysis, extraction, fermentation, and distillation—and particularly on the final product, evaluating quality parameters according to current official standards as well as useful auxiliary parameters in determining the authenticity of the beverage.

## 2. Materials and Methods

### 2.1. Experimental Design

To study the effect of agave age on quality and authenticity parameters of the tequila 100% agave silver class, an experiment was designed at an industrial scale. The experiment consisted of the comparison of the characterization of the product in each of the production stages using agaves of different ages. Each production batch was labeled as “Batch 1”, “Batch 2”, and “Batch 3”, corresponding to the use of agave plants with the ages of 4, 5, and 6 years, respectively. The study was carried out at a tequila company from the Valles region, Jalisco, Mexico (1349 m MSL in average). All of the studies carried out in this investigation were certified and verified by CRT.

The chromatographic analysis was performed in duplicate while the isotope ratio mass spectrometer analysis was performed in triplicate according to the Tequila Regulatory Council recommendations, and the data displayed in the figures and tables correspond to the average value. The statistical analysis consisted of average comparisons of each of the studied parameters, analyzed with one-way analysis of variance (ANOVA) performed in the STATISTICA 10.0 software (Statsoft, Inc., Tulsa, OK, USA), using a *p*-value of 0.05 to determine whether the observed differences are statistically significant.

### 2.2. Industrial-Scale Tequila Production

**Figure 2:**
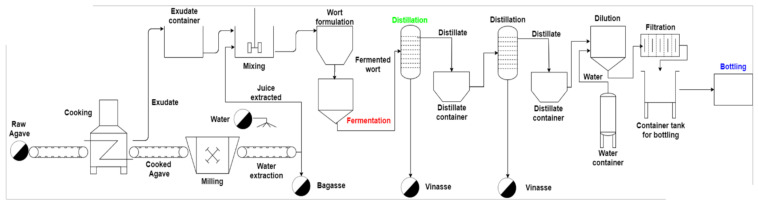
Process diagram for the tequila 100% agave silver class, highlighting in color the stages analyzed in this study.

The tequila production process starts from the plantations in selecting plants that will be harvested as raw material for the elaboration of tequila inside the company. Figure 2 shows a general process diagram of the tequila company where the tequila 100% agave silver class production was carried out. This diagram shows that the main stages required to produce tequila are cooking (hydrolysis), milling, fermentation, distillation, dilution, filtration, and bottling.

#### 2.2.1. Raw Material (*Agave tequilana* Weber Blue Variety)

The *Agave tequilana* Weber blue variety “hearts” (pine without leaves and root) used in this research were cultivated within the DOT in Jalisco, Mexico. The harvested plants were selected according to their age: 4, 5, and 6 years. Before the milling and cooking processes, the morphological characteristics of the plants were measured: height, rosette diameter, and weight. Additionally, the total reducing sugars % (*w*/*w*) and the Brix degrees (°Brix) were determined.

#### 2.2.2. Hydrolysis and Milling Processes

The raw agave hearts are introduced in a masonry oven with a capacity of 30 tons. Once the raw material had been manually loaded, a saturated steam was supplied at a pressure of 4 psi (manometric) until reaching a temperature of 95 °C; then, the steam pressure was lowered to 0.5 psi (manometric) keeping the temperature constant for 36 h until hydrolysis finished. During this stage, the produced exudates (sweet exudate) were drained and separated to mix them after the milling process to obtain the agave juice. This milling process consisted of a conveyor that transports the hydrolyzed agave hearts to a milling train composed of an industrial shredder (the agave fibers are separated), and the agave was squeezed in four three-roller mills to obtain the respective agave juice. The sampling was carried out at the initial and final time of the process.

#### 2.2.3. Fermentation

The yeast used for this research was from the *Saccharomyces cerevisae* species. The fermentation stage consisted of *Agave tequilana* Weber blue variety juices and exudates obtained from the cooking process. The fermentation conditions were as follows: 80:20 agave juices/exudates ratio, 22 °Brix, and an initial pH of 4.5. The fermentation was performed in stainless steel tanks of 30,000 L for 72 h at a regulated temperature of 33 °C in which 0.001 kg of yeast L^−1^ was inoculated. Sampling was carried out at the beginning and the end of this process.

#### 2.2.4. Distillation

The first distillation was performed in a 10,000 L pot still. The second distillation, also known as rectification, was performed on a pot still to eliminate water and to concentrate the alcohol obtained from the previously fermented agave juice. The operating conditions for both the first and second distillations were at a temperature of 95 °C. The product of the first distillation had an alcoholic content of 25–30% (*v*/*v*). Once the second distillation was performed, the alcoholic content increased to 55% (*v*/*v*). Sampling was performed at the end of the first and second distillations.

#### 2.2.5. Final Conditioning and Bottling

Once the second distillation finished, the product was filtered to separate from any solids. The beverage was then bottled and stored. The final product was characterized by evaluating the quality (congeners below the NMX-V-005-NORMEX-2018 established limits) and the proposed authenticity parameters (isotopic ratios).

### 2.3. Chemical Analysis

#### 2.3.1. Total Reducing Sugars (TRS)

The methodology described by NMX-V-006-NORMEX-2019 [20] was used to quantify total reducing sugars using the volumetric method proposed by Lane-Eynon.

#### 2.3.2. Alcoholic Content

Alcoholic content expressed as % ethanol (*v*/*v*) at 20 °C was measured with a DMA-48 density meter (Anton Paar) according to the methodology described by NMX-V-013-NORMEX-2019 [21].

#### 2.3.3. Gas Chromatography

The methanol, higher alcohol, ester, and aldehyde contents were determined according to the methodology described by NMX-V-005-NORMEX-2018 [22]. Moreover, the contents of 2-butanol, n-propanol, 2-methyl-1-propanol, n-butanol, and 3-methyl-1-butanol were determined to retrieve additional information. The analysis was performed on an Agilent 7890B gas chromatograph (Agilent Technologies, Boston, MA USA) with a flame ionization detector and automated sampler with capillary injection. An Agilent J&W DB-WAX UI 30 m by 0.25 mm and 0.25 μm column was used with a backflush system. The furnace was programmed with a temperature ramp starting at 34 °C for 4 min, with increases of 10 °C min^−1^ until a temperature of 160 °C was reached. Then, a second ramp was programmed with increases of 15 °C min^−1^ until 200 °C was reached, and it was kept constant for 3 min. In all cases, a sample volume of 1.0 μL was injected in a split mode with a split ratio of 30:1 using nitrogen as carrier gas with a constant volumetric flow of 1.13 mL min^−1^. Finally, the injection and detection temperatures were set to 250 °C. The detection limits were aldehydes < 4.56 mg/100 mL A.A., furfural < 0.04 mg/100 mL A.A. and esters < 1.98 mg/100 mL A.A.

#### 2.3.4. High-Performance Liquid Chromatography

Furfural was quantified with liquid chromatography using the method described by NMX-V-004-NORMEX-2018 [23], using an Infinity 1260 high-resolution liquid chromatographer (Agilent Technologies, Boston, MA USA). The operating conditions were column Agilent Zorbax XBD-C18 4.6 dimensions 150 mm × 5 μm. The mobile phase was composed of a water-methanol solution (50:50 *w*/*w*, isocratic) with a volumetric flow of 0.5 mL min^−1^. In all cases, the injection volume was 5 μL and a light source adjusted to 280 nm.

#### 2.3.5. Determination of the Isotopic Ratios of Carbon 13 (δ^13^C) and Oxygen 18 (δ^18^O)

Sample conditioning consisted of a distillation process described in the OIV-OENO-426-2011 [24] for the automatic control distillation system. The distillation consisted in collecting the ethanol-water azeotrope at 78 °C with an automated Cadiot column. The water-ethanol azeotrope was completely recovered, with an ethanol composition equal or greater than 92% (*w*/*w*), with a yield of at least 96% to avoid isotope fractionation. Then, the obtained alcohol was analyzed with GC/C/IRMS to determine the isotopic ratios of carbon (δ^13^C) and GC/HTC/IRMS for the isotopic ratios for oxygen (δ^18^O). Firstly, a Trace 1310 gas chromatographer (Thermo Scientific, Waltham, MA, USA) was used to determine the isotopic relations. After the separation, the samples were introduced to Delta V Plus (Thermo Scientific, Waltham, MA, USA), a mass isotope ratio mass spectrometer. Sampling was taken by triplicate, as reported by Fonseca-Aguiñaga et al. (2020) [25].

## 3. Results and Discussion

### 3.1. Analysis of Harvest, Cooking, and Milling Processes

Table 1 shows the physicochemical characterization of agave hearts at different maturation ages. Statistically significant differences were observed in Figure S1a–c (*p* < 0.05), in the morphological data (height, diameter, and weight), which is related to the growth stages of the plant. During the first four years from sowing, the agave develops its growth structures; at this stage, the development of the plant is slow, which is appreciated with slight increases in its weight. However, it is a critical stage to favor good production. From the fourth year, the plant develops considerably, growing exponentially as it begins to store water and sugars, as indicated by the data presented in Table 1, where an increase in weight of 12.5% is observed between four and five years, and 23.18% by weight between five and six years. These results are confirmed with the Total Reducing Sugars (TRS) concentration in which there are statistically significant differences (Figure S1d (*p* < 0.05)), with increases of 39.2% and 25.6% when comparing the data of agaves of five years against four years and six against five years, respectively.

Once the agave hearts were characterized, hydrolysis of the sugars was performed through a cooking stage. In this stage, the first exudates produced during cooking (sour exudate) were separated to eliminate chlorophyll, waxes, fatty acid esters, and undesired odors and flavors.

**Figure 3:**
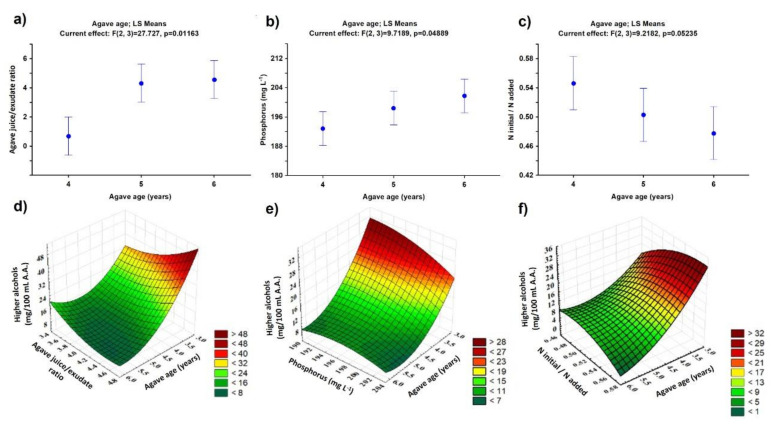
Statistical analysis of the parameters modified during the formulation of must due to agave age: (**a**–**c**) ANOVA analysis; (**d**–**f**) using RSM.

Subsequently, the exudate (sweet exudate) was accumulated to mixed it with the juices extracted in the milling stage to obtain agave fermentation must, with 20 °Bx on average. Due to the low weight of young agave hearts, the yields in the extraction juice are relatively low; for this reason, the juice/exudate ratio was adjusted. Figure 3a shows that there are significant differences for the juice/exudate ratio (*p* < 0.05). Additionally, a correlation has been observed between agave age and nitrogen content in the must. This can be attributed to the nutrient addition to support the plant growth in the early years, where the development of the plant structures is prioritized before sugar production, resulting in a higher nitrogen content in the juice. The above information can be seen more clearly when comparing agaves of 4 vs. 6 years, where the data show statistically significant values.

For this reason, to control the fermentation process, it is necessary to add a nitrogen source using diammonium phosphate (DAP) as a supplementary nutrient for the yeast. The statistical analysis shows that this is not a critical factor in the process since there are no significant differences in their values (Figure 3b (*p* > 0.05)). Nonetheless, the addition of DAP brings, consequently, an unbalanced concentration of phosphorus in the must. Experimental data have shown a statistical difference in this factor (*p* < 0.05) (Figure 3c). When all the variables are analyzed together, it can be concluded that the effect of the juice/exudate ratio has a more significant impact on the process since the *p*-values are much lower (0.011 < 0.048).

### 3.2. Fermentation Stage

**Figure 4:**
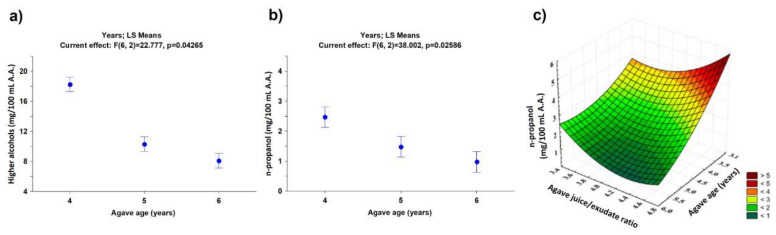
Statistical analysis in the fermentation stage. ANOVA analysis of the effect of agave age on (**a**) the concentration of higher alcohols and (**b**) the concentration of n-propanol; (**c**) analysis using RSM of juice/exudate ratio in the concentration of n-propanol.

Table 2 shows the results obtained from the characterization of congeners generated during the fermentation stage based on the agave age used to prepare the must. The statistical analysis shows that the methanol production is not statistically significant (*p* > 0.05), while higher alcohols have shown a change in their concentration statistically significant (Figure 4a (*p* < 0.05)). It is observed that comparing the content of higher alcohols produced with agaves between 4 vs. 5 and 5 vs. 6 years, there are decreases of 43.6% and 21.8%, respectively, i.e., there is a linear decrease in the higher alcohols with respect to agave age: higher alcohol (mg/100 mL A.A.) = −5.105 (agave age, years) + 37.728, with R^2^ = 0.9056. Additionally, the effect of combining two variables was studied (agave age vs. juice/exudate ratio; agave age vs. phosphorous; and agave age vs. nitrogen ratio) on the higher alcohol production using the Response Surface Methodology (RSM). From the experimental data (Figure 3d–f), mathematical equations were obtained (Equations (1)–(3)), which can estimate these quality parameters in the fermentation stage to produce the tequila 100% agave silver class. This information might be helpful for decision-making to ensure the quality of the final product.

The following correlations were obtained:

HA = 158.45 − 21.66AA − 39.95RA + 4.56(AA)^2^ − 7.12(AA)(RA) + 9.23(RA)^2^, (1)

where HA means higher alcohols (mg/100 mL A.A.), AA is agave age (years), and RA is agave juice/exudate ratio;

HA = −1339.30 − 66.03AA + 15.74P + 2.586(AA)^2^ + 0.18(AA)(P) − 0.04 × P^2^, (2)

where P is phosphorous content (mg L^−1^); and

HA = −342.99 + 0.96AA + 1400.58N_ratio_ + 2.15(AA)^2^ − 53.08(AA)(N_ratio_) − 1091.66(N_ratio_)^2^, (3)

where N_ratio_ is the initial/added nitrogen ratio.

Higher alcohols were analyzed separately to determine the metabolic pathway in which this effect can be observed (see Table 3 for the results). The statistical analysis of the data shows that concentrations of n-propanol and 3-methyl-1-butanol are the congeners that have a statistically significant difference with respect to the age of the agave used in the must (Figure 4b,c, with *p* < 0.05). The content of n-propanol had a lower *p*-value (0.025 < 0.033); hence, this was the congener with the greatest influence in the process since the *p*-value was lower when compared with the other higher alcohols. These results suggest that the n-propanol synthesis during the fermentation is influenced by essential fatty acids (intermediate metabolites), which are important for the development and survival of the microorganism since they contribute to several metabolic pathways as the β-oxidation, promoting the production of intermediates in order to keep the fermentation going [26,27]. The results showed that, in the case of younger agaves, the must formulation has a higher amount of cooking exudates, linked to a higher amount of long odd-chain fatty acids, such as propanoic acid, pentanoic acid, heptanoic acid, and benzoic acid, among others [28]. For this reason, the must is enriched with assimilable fatty acids for the yeast, allowing it to accomplish its metabolic functions, consequently inhibiting the organic acid synthesis route and promoting a metabolic pathway to esters and n-propanol production as described by Eder et al. (2018) [29]. This consideration is based on the increases in these congeners, described in Table 2, where the congeners with a lower content after the fermentation are now more concentrated.

Once it was determined that the juice/exudate ratio significantly affects the generation of higher alcohols, the data were analyzed by a response surface plot (Figure 4c). The results were consistent with Figure 3d, supporting the idea that higher amounts of higher alcohols are linked to the generation of n-propanol. From these data, a mathematical model to estimate this quality parameter in the fermentation stage to produce tequila 100% agave silver class was obtained, which might be helpful to ensure the quality of the product (Equation (4)):

P_OH_ = 26.06 − 0.06AA − 11.00N_ratio_ + 0.26(AA)^2^ − 0.79(AA)(N_ratio_) + 1.81(N_ratio_)^2^ (4)

where P_OH_ is the amount of n-propanol (mg/100 mL A.A.).

### 3.3. Distillation Stage

The results obtained after the distillation process are presented in Table 2. In the first distillation, congeners are enriched because, in this stage, the alcoholic content on the fermented must is separated and concentrated. In the case of the second distillation, the congener content is adjusted to obtain tequila 100% agave silver class as a product.

The presence of esters and aldehydes in the first distillation is due to a higher alcoholic content and congeners in the fermented must. Ethyl acetate has stood out for providing a fruity flavor, being obtained during the fermentation process, having a significant concentration at the time of tequila distillation [30] in which it is important to maintain and regulate the concentration produced in addition to be within the Mexican standard. In the study by Amaya-Delgado et al. (2013) [31], the evaluation of esters was carried out during the tequila fermentation process in which the importance of these within the physicochemical profile was highlighted.

Finally, its production has been related to other congeners during the production process, if there is a positive increase of higher alcohols, esters and ethanol there will be a increase of aldehydes concentration during fermentation process [32]. This has also been correlated with the studies by Arellano et al. (2008) [30], where the fermentation conditions affect the concentration of acetaldehyde because reduction reactions can be carried out to obtain ethanol.

In the second distillation, the amount of the esters was found to be the highest for the batch with 4 years agave. This can be associated with a lower juice/exudate ratio required for younger agaves; thus, a greater amount of exudate is used in the must mix. As previously described, the exudate contains greater amounts of fatty acids (carboxylic acids), which at a higher temperature promote the esterification reaction with the alcohol in solution to produce more esters. The above reaction is favored due to the increase in temperature in this stage. In the case of aldehydes, there are no statistically significant differences between the first and second distillation; the slight appreciable increase may be attributable to the concentration of the analyte due to the second distillation step.

Moreover, the presence of methanol and furfural is associated with the hydrolysis stage (cooking) of the agave hearts, where the demethoxylation and the Maillard reactions are simultaneously promoted at those conditions [17]. Due to the increase in alcoholic content during the distillation process, these congeners can be observed. In the case of methanol, high concentrations prevail in both distillations stages due to its boiling point (64.7 °C), always remaining as part of the light compounds (volatile) known as “*heads*” [19]. Instead, in the case of furfural, its concentration is low in the first distillation, and it can be eliminated in the second due to its high boiling point (161.7 °C) and thus discarded together with all the heavy compounds (less volatile), known as “*tails*”.

The characterization of the higher alcohols is detailed in Table 3. The sum of the total higher alcohols corresponds to the data shown in Table 2. It can be seen that the concentration of the alcohols is correlated to its boiling point at 1 atm: n-propanol (97.1 °C), 2-butanol (99 °C), 2-methyl-1-propanol (108 °C), n-butanol (117.7 °C), and 3-methyl-1-butanol (131 °C); thus a higher concentration of the first congeners is due to a prevalence on the lightweight components in distillation (*heads*), which gives the product a desirable organoleptic profile.

### 3.4. Final Product Analysis

Table 4 and Table 5 show the characterization results of the congeneric compounds found in the final product according to the agave age used in the must. The results show no statistically significant differences, *p* > 0.05 (see Figures S2 and S3), such that in the final conditioning and bottling process, the product shows high-quality parameters regardless of the agave age used in the must; that is, all of the measured values are below the maximum concentrations allowed by the Mexican Official Standard NOM-006-SCFI-2012.

Furthermore, to show the auxiliary parameters of the authenticity in the beverage, mass spectrometry studies of isotopic ratios (δ^13^C_VPDB_ and δ^18^O_VSMOW_) were carried out in the tequila 100% agave silver class samples obtained in this study. The results are presented in Table 4. From this information, it is possible to confirm that the sugar source in beverage production comes from the *Agave tequilana* Weber blue variety since the δ^13^C_VPDB_ and δ^18^O_VSMOW_ are in agreement with previously reported parameters [25,33]. For the case of the δ^13^C_VPDB_ values (−13.41‰ on average), there is no statistically significative difference (*p* > 0.05, Figure S4a) with respect to the agave age used as raw material, since in all cases, the *Agave tequilana* Weber blue variety was used as the only sugar source to produce ethanol. The experimental data show that, for younger agave plants (4 and 5 years), the measured isotopic ratios are more positive values. This effect can be attributed to carbon isotope fractionation during photosynthesis. According with previous reports Hoefs [34] and Park [35], the CO_2_ diffusion process in the agave plant is reversible, while the carbon enzymatic fixation is not:

CO_2,(external)_ ⇌ CO_2,(internal)_ → Organic molecule

Therefore, when the CO_2_ concentration is a limiting factor (as is the case for older agaves), the CO_2_ diffusion to the inner parts of the plant has a limiting step and the carbon isotope fractionation inside the plant decreases. Even though this information is interesting, this behavior should be studied with greater depth to promote the use of this parameter as an auxiliary in determining the agave age used in the process.

On the other hand, the obtained data for the δ^18^O_VSMOW_ (20.53‰ on average) do not show statistically significant differences (*p* > 0.05, Figure S4b) with respect to agave age, thus using this parameter. According to information previously published by Fonseca-Aguiñaga et al. (2021) [36], the distillation process has a correlation with the altitude of the place where it has been carried out because it is related to temperature. As the pressure decreases, with the increase in altitude, the system requires a more significant decrease in temperature to be able to reach the saturated water vapor pressure in such a way that the water molecules constituted by the light isotopes of O and H will stay mostly in the vapor phase concerning the liquid phase; for this reason, the δ^18^O_VSMOW_ of the distillate is enriched. For this reason and considering the altitudes above sea level of the regions of Jalisco in the research work, a mathematical model was proposed to determine the region of origin of production of the beverage from the characterization of δ^18^O_VSMOW_: δ^18^O_VSMOW_ (‰) = −0.0045 × altitude (m MSL) + 26.495, since the experimental data obtained in the present study can be used to validate the estimation of the altitude of the region of origin with an error of 1.7% (altitude (m MSL)_calc_ = ((20.52‰) − 26.495)/−0.0045 = 1327 m MSL). This information can also be useful as an auxiliary parameter to guarantee that the beverage has been produced in the plant and geographical site defined on the manufacturer’s labels from the bottled product that contains the beverage. In this case study, the final product comes from Tequila city, located in the Valles-Jalisco region (altitude (m MSL)_real_ = 1349 m MSL).

## 4. Conclusions

The use of agave plants with different ages (4, 5, and 6 years) as raw material for tequila 100% agave silver class production does not have an impact on the quality parameters since, after the second distillation, the final product has the chromatographic profiles described in the official standard NOM-006-SCFI-2012. The determination of δ^13^C_VPDB_ (−13.41‰) in the final product might be useful as an auxiliary authenticity parameter since it complements the traceability of the raw materials, confirming the type of plant used to produce the beverage. The results of the δ^18^O_VSMOW_ (20.53‰) parameter confirm that it is possible to estimate the altitude of the region where the beverage was produced with an error of 1.7%. This information can be useful as an auxiliary parameter to ensure, from the content of a bottle, if the beverage has been produced from a plant from the geographical location defined in the manufacturer’s label.

The results show that an effective strategy to tackle the scarcity and oversupply of raw material to produce the tequila 100% agave silver class is the use of young agave (4 years). The effect of agave age in the quality parameters of the tequila silver class and other classes will be approached in a future work, paying particular attention to the physicochemical characterization and the sensory profile of the beverage.

## Figures and Tables

**Table 1:** Characterization of agave hearts and must.

Age (Years)	Height (cm)	Diameter (cm)	Average Heart wt. (kg)	TRS (%)	Sugars	Percentage in Must		Nitrogen Content in Juice (mg L^−1^)	
					°Brix	Juice (%)	Exudate (%)	Initial	Added
4	37.5 ± 1.10	41.0 ± 2.20	18.4 ± 0.60	15.21 ± 0.35	19.4 ± 0.60	78.8 ± 0.90	20.9 ± 0.90	95.4 ± 2.80	174.4 ± 2.80
5	40.5 ± 1.60	43.0 ± 1.70	20.7 ± 0.80	18.84 ± 0.45	23.0 ± 0.90	80.9 ± 0.50	18.7 ± 0.50	90.4 ± 1.50	179.4 ± 1.50
6	46.5 ± 2.30	49.5 ± 2.40	25.5 ± 1.30	19.21 ± 0.35	24.2 ± 1.20	81.8 ± 0.70	17.9 ± 0.70	87.4 ± 0.64	182.4 ± 0.60

**Table 2:** Characterization of the obtained congeners during the fermentation and distillation units.

Congeners (mg/100 mL A.A.)	Agave Age (Years)		
	Batch 1 (4 Years Agave)	Batch 2 (5 Years Agave)	Batch 3 (6 Years Agave)
	**Fermentation**		
Methanol	0.08 ± 0.11	1.25 ± 0.40	0.13 ± 0.19
Higher alcohols	18.26 ± 0.26	10.30 ± 0.61	8.05 ± 0.31
Esters	0.00	0.00	0.00
Aldehydes	0.00	0.00	0.00
Furfural	0.00	0.00	0.00
	**First distillation**		
Methanol	573.90 ± 21.96	520.26 ± 8.66	481.06 ± 3.14
Higher alcohols	338.56 ± 18.53	315.12 ± 3.61	294.85 ± 5.90
Esters	11.65 ± 0.13	19.93 ± 1.38	20.41 ± 1.52
Aldehydes	7.36 ± 7.16	1.15 ± 0.28	0.83 ± 0.05
Furfural	0.71 ± 0.08	2.08 ± 0.03	3.36 ± 0.36
	**Second distillation**		
Methanol	244.11 ± 32.75	217.35 ± 17.59	192.19 ± 18.79
Higher alcohols	293.68 ± 7.55	282.54 ± 25.77	247.31 ± 1.25
Esters	34.32 ± 4.75	30.32 ± 12.17	27.03 ± 5.05
Aldehydes	4.76 ± 0.11	3.53 ± 0.94	3.25 ± 0.04
Furfural	0.42 ± 0.12	0.51 ± 0.04	0.34 ± 0.04

**Table 3:** Characterization of higher alcohols in the fermentation and distillation stages.

Higher Alcohol (mg/100 mL A.A.)	Agave Age (Years)		
	Batch 1 (4 Years Agave)	Batch 2 (5 Years Agave)	Batch 3 (6 Years Agave)
**Fermentation**			
2-butanol	0.00	0.00	0.00
n-propanol	2.47 ± 0.18	1.48 ± 0.09	0.97 ± 0.16
2-methyl-1-propanol	4.85 ± 0.37	4.08 ± 0.19	3.82 ± 0.09
n-butanol	0.00	0.00	0.00
3-methyl-1-butanol	10.94 ± 0.30	4.75 ± 0.32	3.29 ±0.06
**First distillation**			
2-butanol	0.00	0.00	0.00
n-propanol	90.83 ± 0.17	78.94 ± 0.60	79.00 ± 1.66
2-methyl-1-propanol	69.86 ± 1.76	65.55 ± 0.75	64.58 ± 0.87
n-butanol	0.00	0.00	0.00
3-methyl-1-butanol	177.87 ± 16.60	170.63 ± 3.77	151.27 ± 3.37
**Second distillation**			
2-butanol	0.62 ± 0.12	0.54 ± 0.13	0.18 ± 0.08
n-propanol	61.21 ± 0.40	58.32 ± 5.45	46.04 ± 3.39
2-methyl-1-propanol	63.19 ± 4.36	57.18 ± 4.09	52.63 ± 0.91
n-butanol	0.73 ± 0.11	0.69 ± 0.21	0.54 ± 0.11
3-methyl-1-butanol	167.43 ± 11.31	165.25 ± 15.88	147.42 ± 1.27

**Table 4:** Congener characterization of the final product (tequila 100% agave silver class) and additional authenticity parameters.

Age (years)	Methanol (mg/100 mL A.A.)	Higher Alcohols (mg/100 mL A.A.)	Esters (mg/100 mL A.A.)	Aldehydes (mg/100 mL A.A.)	Furfural (mg/100 mL A.A.)	δ ^13^C_VPDB_ (‰)	δ ^18^O_VSMOW_ (‰)
4	244.11 ± 32.75	293.68 ± 7.55	34.32 ± 4.75	4.76 ± 0.11	0.42 ± 0.12	−13.37 ± 0.05	20.91 ± 1.72
5	217.35 ± 17.59	282.54 ± 25.77	30.32 ± 12.17	3.53 ± 0.94	0.51 ± 0.04	−13.36 ± 0.13	20.11 ± 0.81
6	192.19 ± 18.79	247.31 ± 1.25	27.03 ± 5.05	3.25 ± 0.04	0.34 ± 0.04	−13.49 ± 0.17	20.56 ± 1.25

**Table 5:** Characterization of higher alcohols in the final product (tequila 100% agave silver class).

Age (years)	2-Butanol (mg/100 mL A.A.)	N-Propanol (mg/100 mL A.A.)	2-Methyl-1-propanol (mg/100 mL A.A.)	1-Butanol (mg/100 mL A.A.)	3-Methyl-1-butanol (mg/100 mL A.A.)
4	0.62 ± 0.12	61.21 ± 0.40	63.19 ± 4.36	0.73 ± 0.11	167.43 ± 11.31
5	0.54 ± 0.13	58.32 ± 5.45	57.18 ± 4.09	0.69 ± 0.21	165.25 ± 15.88
6	0.18 ± 0.08	46.04 ± 3.39	52.63 ± 0.91	0.54 ± 0.11	147.42 ± 1.27

## Data Availability

The data that support the findings of this study are available from the corresponding author upon reasonable request.

## Supplementary Materials

The following are available online at https://www.mdpi.com/article/10.3390/foods10123103/s1, Figure S1: Statistical analysis of the physicochemical characterization of the agave hearts from different ages. Figure S2: Statistical analysis of the congeners in the final product (tequila 100% agave silver class). Figure S3: Statistical analysis of higher alcohols in the final product (tequila 100% agave silver class). Figure S4: Statistical analysis of isotopic ratio of the final product: (a) δ^13^C_VPDB_ (‰), (b) δ ^18^O_VSMOW_ (‰) (tequila 100% agave silver class).
