# Supplementary material for: Effect of Age of Agave tequilana Weber Blue Variety on Quality and Authenticity Parameters for the Tequila 100% Agave Silver Class: Evaluation at the Industrial Scale Level
Source: Foods. 2021 Dec 14;10(12):3103. doi: 10.3390/foods10123103 (PMC8701365; doi:10.3390/foods10123103)
Supplement: Supplementary file 1 [file foods-10-03103-s001.zip › foods-1471580-supplementary.pdf]

Article

Effect of Agave *tequilana* Weber blue variety age on quality and authenticity parameters of the Tequila 100% agave silver class: evaluation at the industrial scale level.

Efraín Acosta-Salazar [1], Rocío Fonseca-Aguiñaga [2,3] , Walter M. Warren-Vega [2] , Ana I. Zárate-Guzmán [2,4] , Marco A. Zárate-Navarro [1], Luis A. Romero-Cano [2,*], Armando Campos-Rodríguez [1,*]

[1] Departamento de Ciencias Biotecnológicas y Ambientales. Universidad Autónoma de Guadalajara. Av. Patria 1201, C.P. 45129. Zapopan, Jalisco. México.
[2] Grupo de Investigación en Materiales y Fenómenos de Superficie. Departamento de Ciencias Biotecnológicas y Ambientales. Universidad Autónoma de Guadalajara. Av. Patria 1201, C.P. 45129. Zapopan, Jalisco. México
[3] Laboratorio de Isotopía, Consejo Regulador del Tequila A. C. Av. Patria 723, Zapopan, Jalisco C.P. 45030, México
[4] Centro de Investigación y Estudios de Posgrado, Facultad de Ciencias Químicas, Universidad Autónoma de San Luis Potosí, San Luis Potosí 78060, México
* Correspondence: armando.crodriguez@edu.uag.mx (A.C.-R.); luis.romero@edu.uag.mx (L.A.R.-C.)

Supplementary Materials

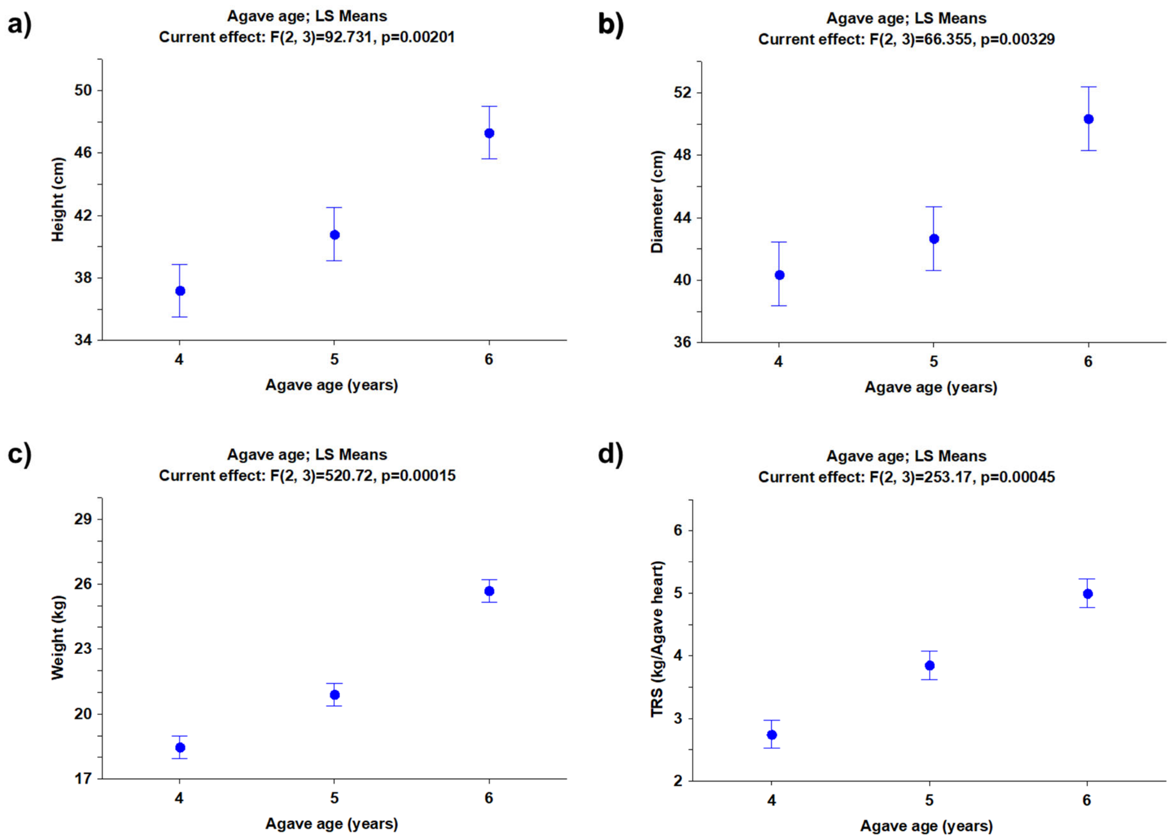

Figure S1. Statistical analysis of the physicochemical characterization of the agave hearts from different ages: (**a**)Height (cm), (**b**) Diameter (cm), (**c**) Weight (kg), (**d**) TRS (kg/Agave heart).

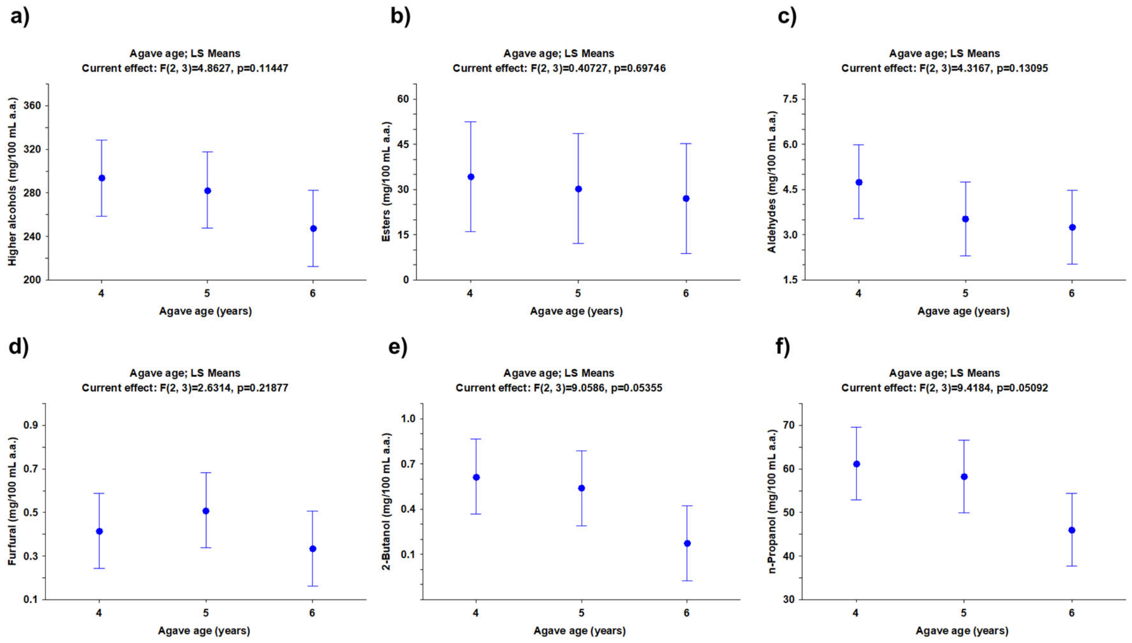

Figure S2. Statistical analysis of the congeners in the final product (tequila 100% agave silver class): (**a**) Higher alcohols (mg/100 mL a.a.), (**b**) Esters (mg/100 mL a.a.), (**c**) Aldehydes (mg/100 mL a.a.), (**d**) Furfural (mg/100 mL a.a.), (**e**) 2-Butanol (mg/100 mL a.a.), n-Propanol (mg/100 mL a.a.).

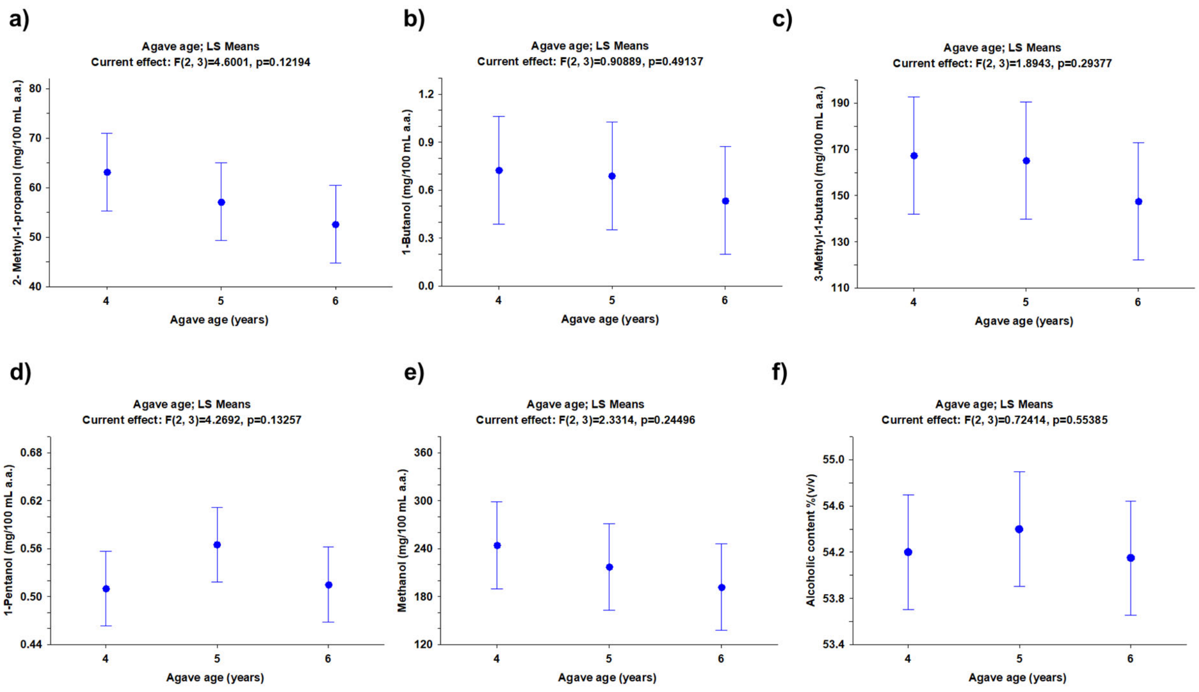

Figure S3. Statistical analysis of higher alcohols the final product (tequila 100% agave silver class): (**a**) 2-Methyl-1-propanol (mg/100 mL a.a.), (**b**) 1-Butanol (mg/100 mL a.a.), (**c**) 3-Methyl-1-butanol (mg/100 mL a.a.), (**d**) 1-Pentanol (mg/100 mL a.a.), (**e**) Methanol (mg/100 mL a.a.), alcoholic content %(V/V).

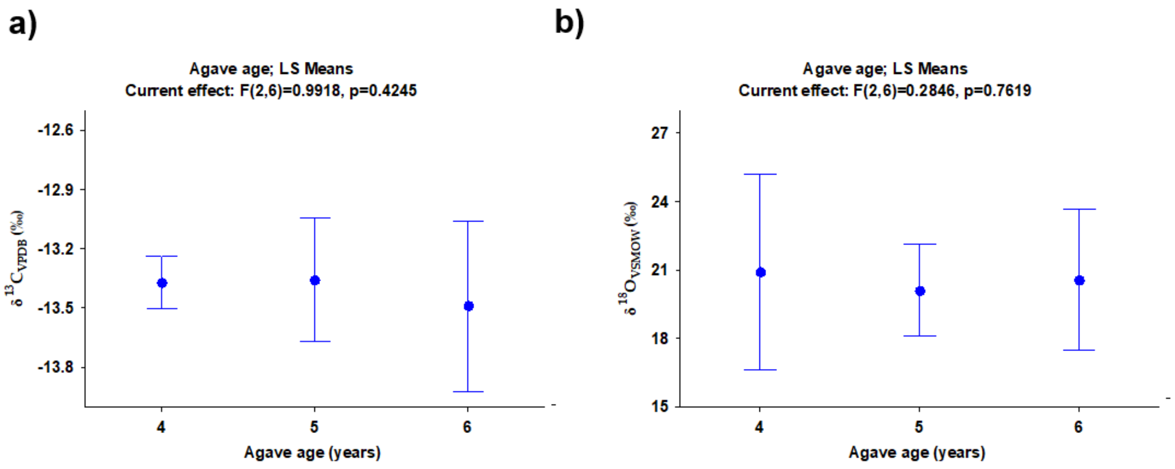

Figure S4. Statistical analysis of isotopic ratio of the final product: (**a**) δ^{13}**C**$_{VPDB}$ **(‰),** (**b**) δ^{18}**O**$_{VSMOW}$ **(‰)** (tequila 100% agave silver class).
